# A230 SARCOPENIA IN PRIMARY SCLEROSING CHOLANGITIS AND INFLAMMATORY BOWEL DISEASE IN PAEDIATRICS AND CLINICAL IMPLICATIONS

**DOI:** 10.1093/jcag/gwad061.230

**Published:** 2024-02-14

**Authors:** G Dahlwi, T Yodoshi, P Church, A Ricciuto

**Affiliations:** Division of Gastroenterology, Hepatology and Nutrition, The Hospital for Sick Children Department of Paediatrics, Toronto, ON, Canada; Division of Gastroenterology, Hepatology and Nutrition, The Hospital for Sick Children Department of Paediatrics, Toronto, ON, Canada; Division of Gastroenterology, Hepatology and Nutrition, The Hospital for Sick Children Department of Paediatrics, Toronto, ON, Canada; Division of Gastroenterology, Hepatology and Nutrition, The Hospital for Sick Children Department of Paediatrics, Toronto, ON, Canada

## Abstract

**Background:**

Sarcopenia is defined in paediatrics as a decrease in muscle mass for a given age. The coexistence of primary sclerosing cholangitis (PSC) and inflammatory bowel disease (IBD) is recognized to be a distinct entity with a distinct phenotype. However, to date, there is no study that has assessed the prevalence of sarcopenia and its clinical implications in paediatric PSC-IBD, compared to paediatric colitis.

**Aims:**

We first aimed to determine whether sarcopenia is more prevalent in paediatric PSC-IBD than children with colitis without liver disease. Our secondary aim was to explore the prognostic implications of sarcopenia in paediatric PSC-IBD both for liver and IBD outcomes.

**Methods:**

This was a single-center retrospective cohort study including paediatric IBD patients with and without PSC who underwent magnetic resonance enterography (MRE) for clinical care. Psoas muscle area (PMA) at the L4-5 intervertebral disc level was calculated. Sarcopenia was defined as PMA ampersand:003C 3^rd^ percentile for age and sex. The association between PSC and sarcopenia was examined using logistic regression, adjusting for variables selected using change in estimate method. Survival analysis was used to examine the association between sarcopenia and IBD/liver outcomes.

**Results:**

A total of 85 IBD patients were included – 31 PSC-IBD and 54 colitis controls. Both groups had male predominance. A higher proportion of children with PSC-IBD had pancolitis (Pampersand:003C0.001), and PSC-IBD patients had less severe clinical IBD disease activity than controls (P=0.003). Sarcopenia was present in 39% PSC-IBD and 18% colitis (odds ratio (OR) 2.7, 95%CI 1.02-7.53, P=0.044). Adjusting for endoscopic Mayo score and biologic therapy exposure prior to MRE, the adjusted OR for the association between PSC and sarcopenia was 4.22, 95%CI 1.32-13.44, P=0.015. Sarcopenic patients were younger than non-sarcopenic patients (mean 12.4(SD2.9) years versus 14.3(SD2.3) years, P=0.003), and also had lower weight and height (Pampersand:003C0.001, P=0.002, respectively). Sarcopenia was not a risk factor for escalation to biologics and/or surgery (hazards ratio (HR) 1.18, 95%CI 0.55-2.50, P=0.67) nor for progression to liver fibrosis and/or portal hypertension (HR 0.40, 95%CI 0.044-3.54, P=0.40).

**Conclusions:**

Sarcopenia was 4 times more frequent in PSC-IBD compared to colitis controls in our cohort, after accounting for differences in IBD severity and biologic exposure. Sarcopenia may represent an additional feature that is distinct to PSC-IBD versus colitis without associated PSC.

**Funding Agencies:**

None

